# Atherogenic index of plasma and risk of diabetic nephropathy in type 2 diabetes: A meta-analysis

**DOI:** 10.17305/bb.2025.12731

**Published:** 2025-08-01

**Authors:** Danyan Min, Junli Zhao, Miao Liu

**Affiliations:** 1 Department of Nephrology, Shanghai University of Medicine and Health Sciences Affiliated Zhoupu Hospital, Shanghai, China

**Keywords:** Type 2 diabetes, atherogenic index of plasma, diabetic nephropathy, proteinuria, meta-analysis

## Abstract

The atherogenic index of plasma (AIP) is a lipid-based biomarker associated with cardiovascular and renal risks in individuals with type 2 diabetes mellitus (T2DM). However, its relationship with diabetic nephropathy (DN) remains inadequately defined. This meta-analysis aims to assess the association between AIP and DN in T2DM patients. We conducted a comprehensive search in PubMed, Embase, and Web of Science for observational studies that compared the incidence or prevalence of DN across varying AIP levels in T2DM populations. Data were synthesized using a random-effects model to account for potential heterogeneity. A total of eleven datasets from ten studies, encompassing 25,773 T2DM patients, were included in the analysis. The pooled results indicated that higher AIP levels are significantly associated with DN (risk ratio [RR] ═ 1.51, 95% confidence interval [CI]: 1.36–1.67; *P* < 0.001). Subgroup analyses revealed a stronger association in patients aged 58 years or older (RR = 1.66) compared to those younger than 58 years (RR = 1.35; *P* for subgroup difference = 0.02). Similar associations were observed across different study designs, sex distributions, AIP cutoff values, definitions of DN, and quality scores (*P* for subgroup difference all > 0.05). Meta-regression analysis further indicated that older age positively influenced the strength of the association (coefficient = 0.018, *P* ═ 0.03). In conclusion, elevated AIP levels are significantly associated with DN in T2DM patients, particularly among older individuals.

## Introduction

Diabetic nephropathy (DN) is a significant microvascular complication of type 2 diabetes mellitus (T2DM), characterized by persistent albuminuria, decreased glomerular filtration rate (GFR), and an elevated risk of end-stage kidney disease (ESKD) [1, 2]. It ranks among the leading causes of chronic kidney disease (CKD) and dialysis globally, responsible for nearly half of all new ESKD cases [3]. The prevalence of DN continues to escalate alongside the global diabetes epidemic, with projections indicating that up to 40% of T2DM patients will experience renal complications during their illness [4]. DN not only diminishes quality of life and increases healthcare costs but also significantly elevates the risk of cardiovascular morbidity and all-cause mortality [5, 6]. Despite progress in managing glycemia and blood pressure, the onset and progression of DN remain inadequately preventable, highlighting the urgent need to identify early and reliable risk factors to facilitate timely intervention and risk stratification [7].

The atherogenic index of plasma (AIP), defined as the base-10 logarithm of the ratio of triglycerides (TG) to high-density lipoprotein cholesterol (HDL-C), serves as an indicator of the balance between atherogenic and protective lipid fractions [8, 9]. Elevated AIP levels are recognized as a surrogate marker for small dense low-density lipoprotein particles and have been linked to endothelial dysfunction, oxidative stress, and insulin resistance—mechanisms that contribute to the pathogenesis of DN [10, 11]. Clinically, AIP is utilized as a straightforward and cost-effective biomarker for assessing cardiovascular and metabolic risk in patients with T2DM [12, 13]. Recent studies have increasingly focused on the potential relationship between AIP and renal complications, particularly DN [14, 15]. However, existing research presents inconsistent findings, with some studies indicating a robust association [16–23] while others report no significant correlation [24, 25]. Given the growing clinical importance of AIP and the urgent need to identify novel predictors of DN, we conducted a meta-analysis to systematically assess the association between AIP and the risk of DN in individuals with T2DM.

## Materials and methods

This meta-analysis was conducted in accordance with the PRISMA 2020 statement [26, 27] and the Cochrane Handbook for Systematic Reviews [27]. These guidelines informed the development of the protocol, data collection, statistical synthesis, and reporting procedures. The protocol has been prospectively registered in the International Prospective Register of Systematic Reviews (PROSPERO) database under the identifier CRD420251061587.

### Database search

To identify studies relevant to this meta-analysis, we conducted a comprehensive search of the PubMed, Embase, and Web of Science databases using a broad range of search terms, including: (1) “atherogenic index of plasma” OR “atherogenic index” OR “AIP”; (2) “diabetes” OR “diabetic”; (3) “renal” OR “kidney” OR “nephropathy” OR “proteinuria” OR “albuminuria” OR “nephropathies.” The literature search was restricted to studies involving human participants and included only full-length, peer-reviewed articles published in English. This limitation was implemented to align with the language of the journals’ readership and to ensure the inclusion of studies with consistent methodological quality. Numerous high-quality studies from East Asia are published in English-language journals indexed in the selected databases. We excluded Chinese-language articles from regional databases to avoid an incomplete representation of non-English literature from other regions and to mitigate potential language or regional bias. Grey literature, including preprints, dissertations, and conference abstracts, was also excluded due to concerns regarding the absence of peer review, potential data incompleteness, and unclear methodological rigor. To ensure comprehensive coverage, we manually screened the reference lists of pertinent original and review articles for additional eligible studies. The search extended from the inception of each database until May 6, 2025, with the full search strategies detailed in Supplemental File 1.

### Study selection

The inclusion criteria were structured according to the Population, Intervention, Comparison, Outcome and Study design (PICOS) framework.

Population (P): Adults aged 18 years or older with a confirmed diagnosis of T2DM.

Exposure (I): Patients with a high AIP were classified as the exposure group. AIP is calculated as the base-10 logarithm of the ratio of triglycerides (TG) to HDL-C, with both TG and HDL-C expressed in mmol/L. The methods and cutoffs for defining a high AIP were consistent with those used in the original studies [16–25].

Comparison (C): Patients with a low AIP.

Outcome (O): The incidence or prevalence of DN was compared between T2DM patients in the highest and lowest categories of AIP. The diagnosis and validation of DN in these patients adhered to the criteria established in the original studies, which typically included persistent albuminuria (defined as a urinary albumin-to-creatinine ratio [UACR] ≥ 30 mg/g), a reduced estimated glomerular filtration rate (eGFR < 60 mL/min/1.73 m^2^) in the absence of other primary kidney diseases, or a clinical diagnosis of nephropathy attributed to diabetes. Although the term “diabetic kidney disease” (DKD) is now more commonly used to refer to diabetes-related renal impairment broadly, we employed the term DN to align with the terminology used in the included studies [3].

Study design (S): Observational studies, including prospective or retrospective cohort studies, cross-sectional studies, and case-control studies.

Exclusion criteria: Reviews, editorials, meta-analyses, preclinical studies, and studies that included non-diabetic patients, did not evaluate AIP as an exposure, or failed to report the incidence or prevalence of DN were excluded. In instances of overlapping populations, the study with the most comprehensive dataset was included.

### Study quality evaluation and data collection

The literature search, study selection, quality assessment, and data extraction were conducted independently by two reviewers, with any disagreements resolved through discussion with the corresponding author. Study quality was evaluated using the Newcastle–Ottawa Scale (NOS), which assesses three domains: participant selection, control for confounding, and outcome assessment [28]. The NOS assigns scores from 1 to 9, with higher scores indicating greater quality; studies scoring 7 or above were classified as high quality. Extracted data encompassed study-level information (first author, publication year, country, and study design), participant characteristics (number of patients with T2DM, mean age, and sex distribution), methods for determining the cutoff of AIP and cutoff values for defining a high AIP in each study, median follow-up durations for cohort studies, diagnostic methods for DN, numbers of patients with prevalent or newly developed DN, and the covariates adjusted for in the association analyses.

### Statistical analysis

The relationship between AIP and DN in patients with T2DM was assessed by pooling risk ratios (RRs) and their corresponding 95% confidence intervals (CIs). This analysis compared T2DM patients with high AIP levels to those with low AIP levels. In instances where studies reported hazard ratios (HRs), these were considered equivalent to RRs due to their similar interpretative value for time-to-event outcomes [29]. For studies that presented odds ratios (ORs), these were converted to RRs using the formula: RR = OR/(1 -- pRef + pRef × OR), where pRef represents the prevalence of DN in the reference group (i.e., the low AIP group) [30]. This conversion method has been validated in previous meta-analyses. When necessary, RRs and their standard errors were derived from reported 95% CIs or *P* values, followed by log transformation to stabilize variance and normalize the distribution [27]. Between-study heterogeneity was evaluated using the Cochrane *Q* test and the *I*^2^ statistic, with interpretations of low (< 25%), moderate (25%–75%), and high (> 75%) heterogeneity [31]. A random-effects model was employed to accommodate the anticipated variation across studies [27].

Sensitivity analysis was conducted by sequentially omitting individual studies to assess the stability of the pooled estimate. Additionally, a sensitivity analysis limited to cohort studies was performed to validate the findings. Univariate meta-regression analyses were conducted to evaluate the influence of study characteristics on continuous variables related to the association between AIP and DN. These characteristics included the mean patient ages, proportions of male participants, cutoff values for high AIP levels, and NOS scores [27]. Subgroup analyses were also undertaken to investigate the impact of study-level characteristics, such as patient age, study design, male proportions, AIP cutoff values, diagnostic criteria for DN, and NOS scores of the included studies [27]. The median values across the included studies served as cutoff points for continuous subgroup variables including age, male proportion, AIP cutoff value, and NOS score. For age, the median of 58.7 years was rounded down to 58 years for stratification purposes. Publication bias was assessed through visual inspection of funnel plots and formally tested using Egger’s regression test [32]. To further evaluate the potential impact of missing or unpublished studies, we employed Duval and Tweedie’s trim-and-fill analysis [33]. This method estimates the number of potentially missing studies and recalculates an adjusted pooled effect size by imputing these studies [33]. A *P* value of < 0.05 was considered statistically significant. All statistical analyses were performed using RevMan (version 5.1; Cochrane Collaboration, Oxford, UK) and Stata (version 12.0; Stata Corporation, College Station, TX, USA).

### Certainty of evidence

The certainty of evidence for the primary outcome was evaluated using the GRADE (Grading of Recommendations, Assessment, Development, and Evaluation) framework [34]. This assessment encompassed five domains: study limitations, inconsistency, indirectness, imprecision, and publication bias. Each outcome was categorized as high, moderate, low, or very low certainty [34]. A Summary of Findings table was subsequently generated to illustrate these results.

## Results

### Study retrieval

**Figure 1.**
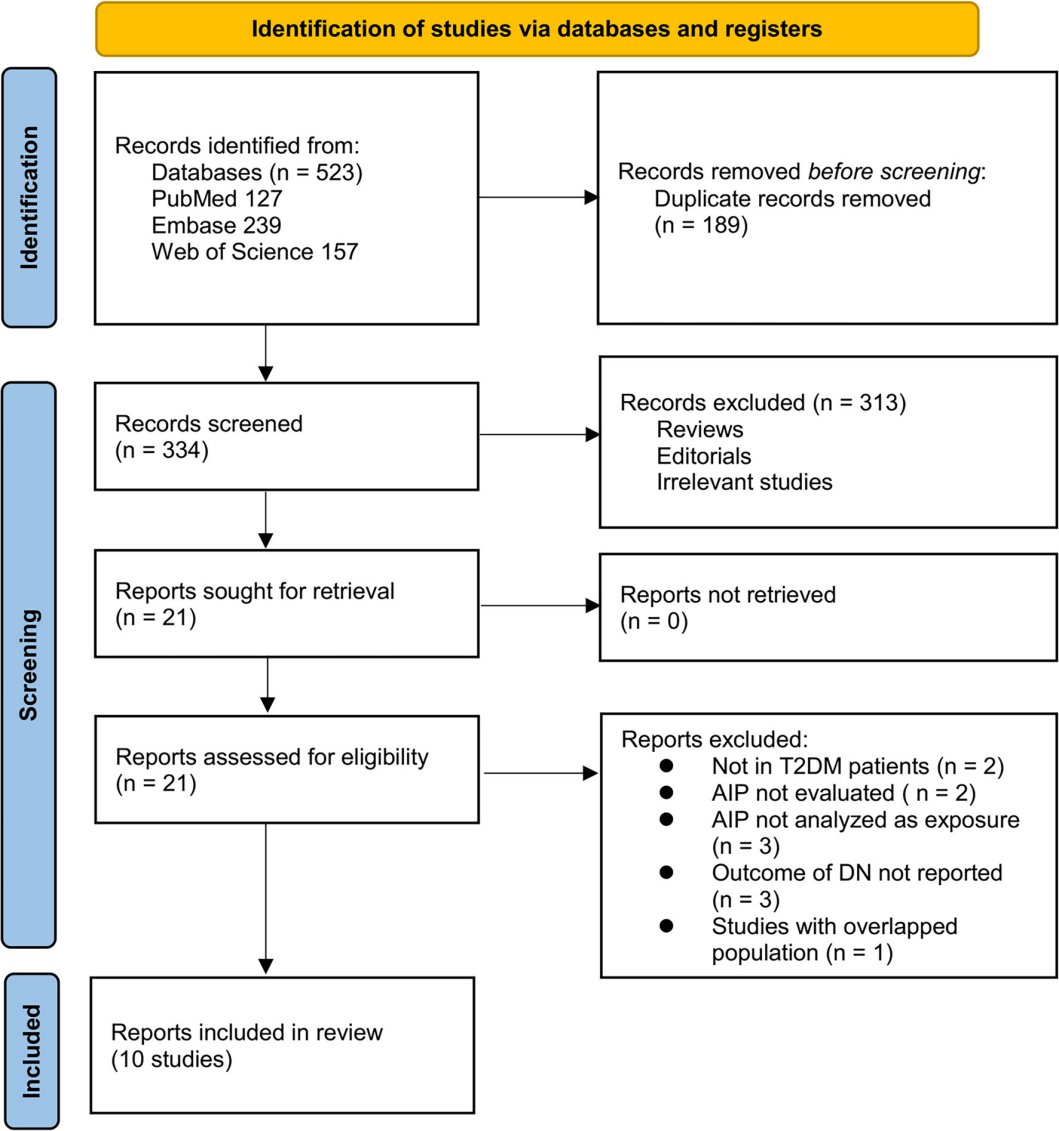
**Flow diagram of study selection process.** The diagram illustrates the number of records identified, screened, assessed for eligibility, and included in the final meta-analysis, following the PRISMA 2020 guidelines.

The study selection process is illustrated in Figure 1. Initially, 523 potentially relevant records were identified through database searches and citation screening. After removing 189 duplicates, 334 records remained for title and abstract screening, resulting in the exclusion of 313 articles that did not align with the objectives of the meta-analysis. The full texts of the remaining 21 articles were independently assessed by two reviewers, leading to the exclusion of 11 studies for reasons detailed in Figure 1. Ultimately, ten studies met the inclusion criteria and were incorporated into the quantitative synthesis [16–25].

### Overview of the study characteristics

Table 1 presents the characteristics of the ten studies included in this meta-analysis [16–25], published between 2022 and 2025, and conducted in China, Iran, Korea, and the United States. One publication [23] reported independent data from two distinct populations in the US and Korea, treated as separate datasets for the meta-analysis. Consequently, 11 datasets from 10 studies were available. Among these, seven were cross-sectional studies [16–18, 21–23, 25] and three were prospective cohort studies [19, 20, 24], encompassing a total of 25,773 patients with T2DM. The mean age of participants ranged from 53.8 to 63.4 years, with the proportion of male patients varying between 43.7% and 67.7%. AIP was categorized using previously defined cutoffs [17], medians [21], tertiles [19, 22, 25], or quartiles [16, 18, 20, 23, 24], with cutoff values for high AIP levels ranging from 0.15 to 0.51. The follow-up durations for the three prospective cohort studies varied from 2 to 6 years. DN was diagnosed based on UACR ≥ 30 mg/g [16, 18, 22, 23], eGFR < 60 mL/min/1.73 m^2^ [17, 20, 24], or both [19, 21, 25]. The number of DN cases across studies ranged from 53 to 1572, with a total of 6934 (26.9%) patients diagnosed with DN. All studies conducted multivariate adjustments, typically including age, sex, body mass index (BMI), blood pressure, glycemic markers, lipid profiles, and comorbidities. As illustrated in Table 2, the NOS scores of the included studies were 8 or 9, indicating overall good methodological quality.

### Association between AIP and DN

**Figure 2.**
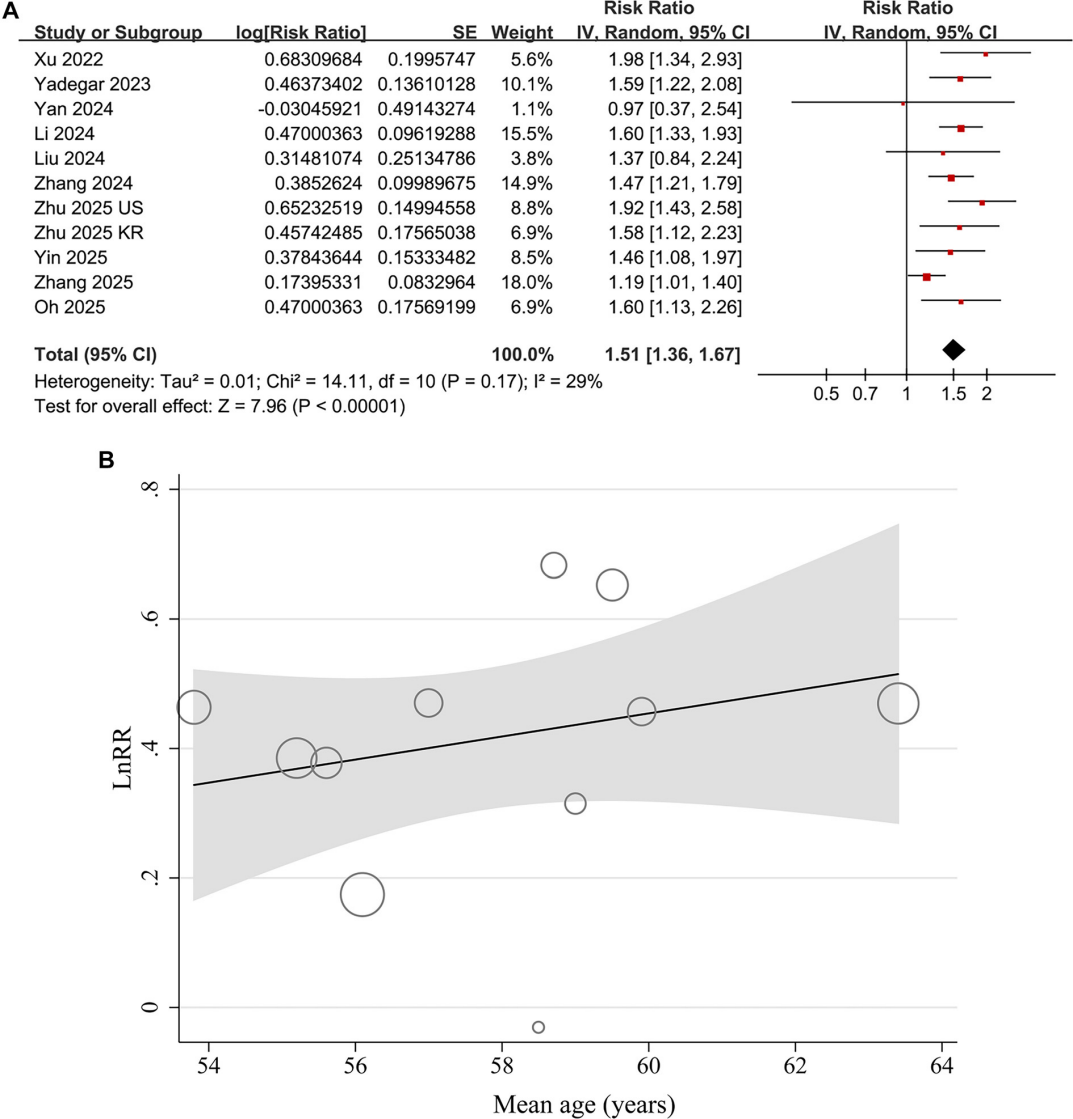
**Forest plots and meta-regression analysis of the association between AIP and DN in patients with T2DM.** (A) Forest plot for the overall meta-analysis of the association between a high AIP and the risk of DN, using risk ratios after metric conversion; (B) Meta-regression analysis for the influence of mean age on the association between AIP and DN. This analysis is exploratory in nature due to the limited number of data points (*n* ═ 11). Abbreviations: AIP: Atherogenic index of plasma; DN: Diabetic nephropathy; T2DM: Type 2 diabetes mellitus.

A pooled analysis of 11 datasets derived from ten studies [16–25] demonstrated a significant association between a high AIP was significantly associated with DN in patients with T2DM (RR = 1.51, 95% confidence interval [CI]: 1.36–1.67; *P* < 0.001; Figure 2A). Moderate heterogeneity was observed across the studies (*I*^2^ ═ 29%). Sensitivity analyses, conducted by excluding one study at a time, yielded consistent results (RR range: 1.47–1.58, all with *P* < 0.05). Furthermore, a sensitivity analysis restricted to three cohort studies [19, 20, 24] also indicated consistent findings (RR = 1.49, 95% CI: 1.27–1.75; *P* < 0.001; *I*^2^ ═ 0%).

Univariate meta-regression analyses revealed a positive correlation between the mean age of patients and the relationship between AIP and DN in T2DM patients (coefficient = 0.018, *P* ═ 0.03; Table 3 and Figure 2B), which accounted for a substantial portion of the heterogeneity across studies (Adjusted *R*^2^ ═ 24.3%). Other variables, such as the proportion of male participants, AIP cutoff values, and NOS scores, did not significantly influence the association between AIP and DN (all *P* > 0.05; Table 3).

**Figure 3.**
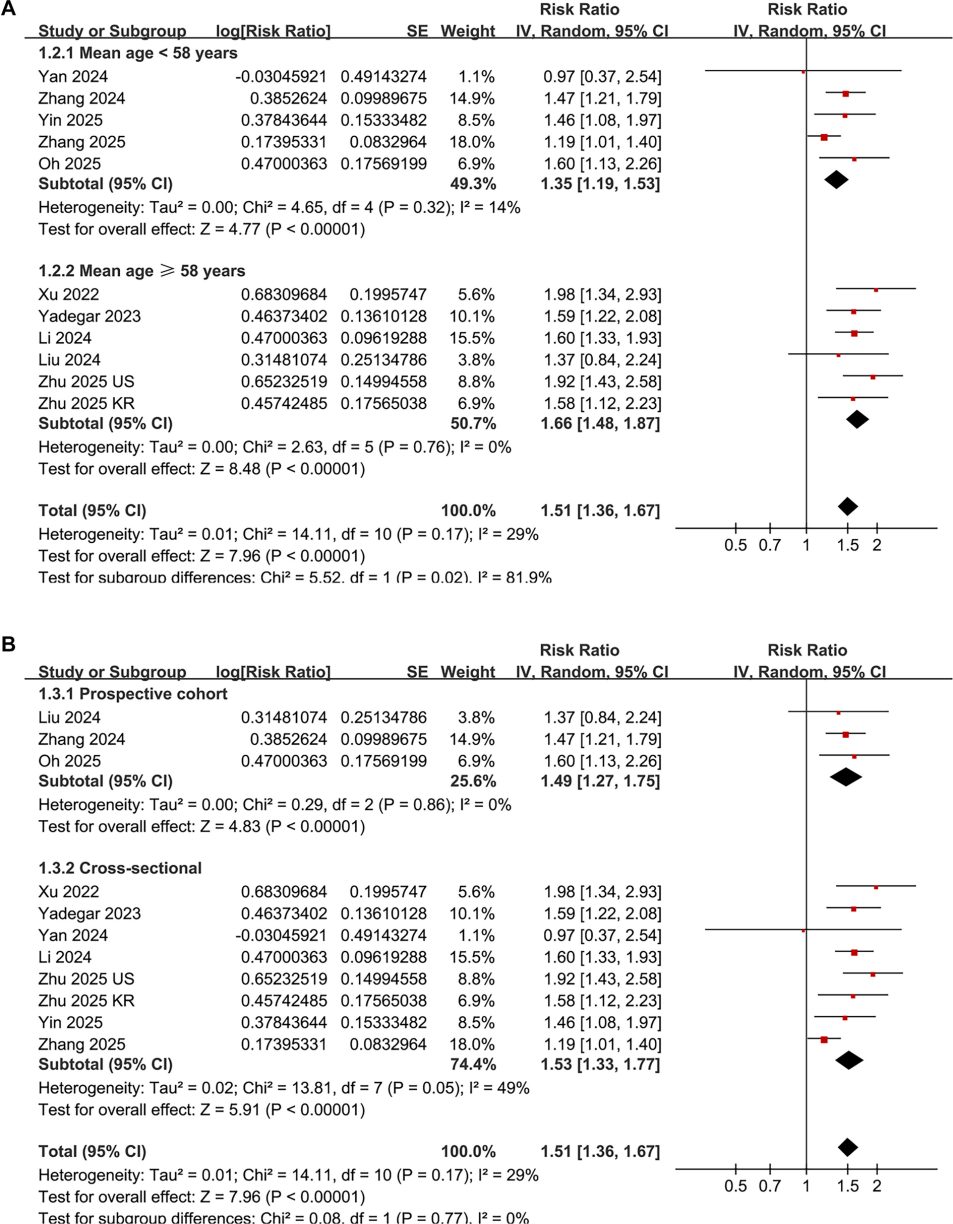
**Subgroup analyses of the association between AIP and DN in patients with T2DM.** (A) Subgroup analysis stratified by mean age of the study population (<58 vs ≥58 years; based on a median value of 58.7 years, rounded down for stratification); (B) Subgroup analysis based on study design (cross-sectional vs prospective cohort). Risk ratios (RRs) and 95% confidence intervals (CIs) are presented for each subgroup. Abbreviations: AIP: Atherogenic index of plasma; DN: Diabetic nephropathy; T2DM: Type 2 diabetes mellitus.

**Figure 4.**
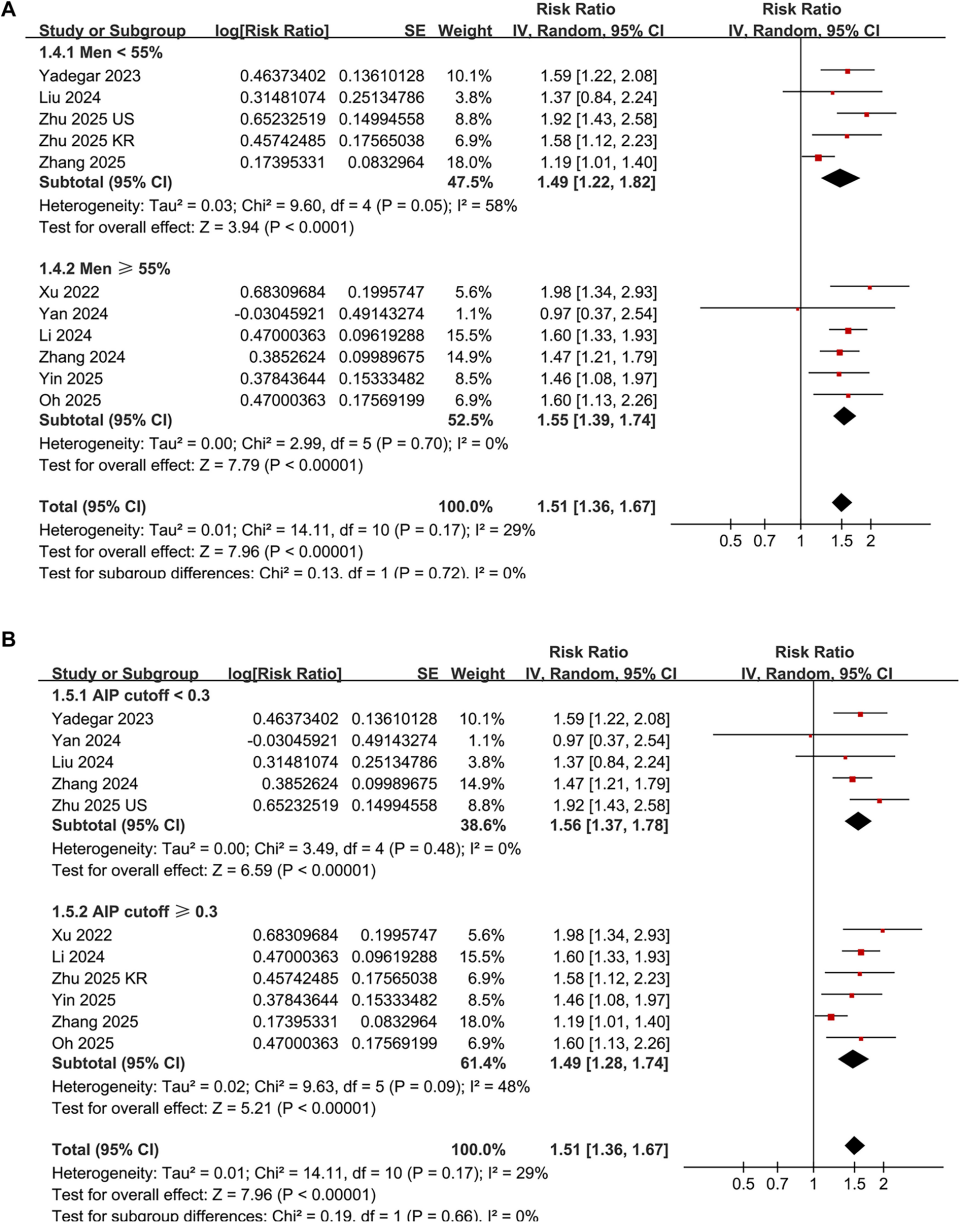
**Subgroup analyses of the association between AIP and DN in patients with T2DM.** (A) Subgroup analysis based on the proportion of male participants in the study population (<55% vs ≥55%); (B) Subgroup analysis based on AIP cutoff values used to define high versus low AIP (<0.3 vs ≥0.3). Pooled risk ratios (RRs) and 95% confidence intervals (CIs) are shown for each subgroup.

Additionally, subgroup analyses revealed a stronger association between elevated AIP and DN in patients aged 58 years or older compared to those younger than 58 years (RR: 1.66 vs 1.35, *P* for subgroup difference = 0.02; Figure 3A). Subsequent subgroup analyses showed comparable results in both prospective cohort and cross-sectional studies (RR: 1.49 vs 1.53, *P* for subgroup difference = 0.77; Figure 3B), in studies with male proportions less than or equal to 55% vs those greater than or equal to 55% (RR: 1.49 vs 1.55, *P* for subgroup difference = 0.72; Figure 4A), and in studies utilizing AIP cutoff values less than or equal to 0.3 vs those greater than or equal to 0.3 (RR: 1.56 vs 1.49, *P* for subgroup difference = 0.66; Figure 4B).

**Figure 5.**
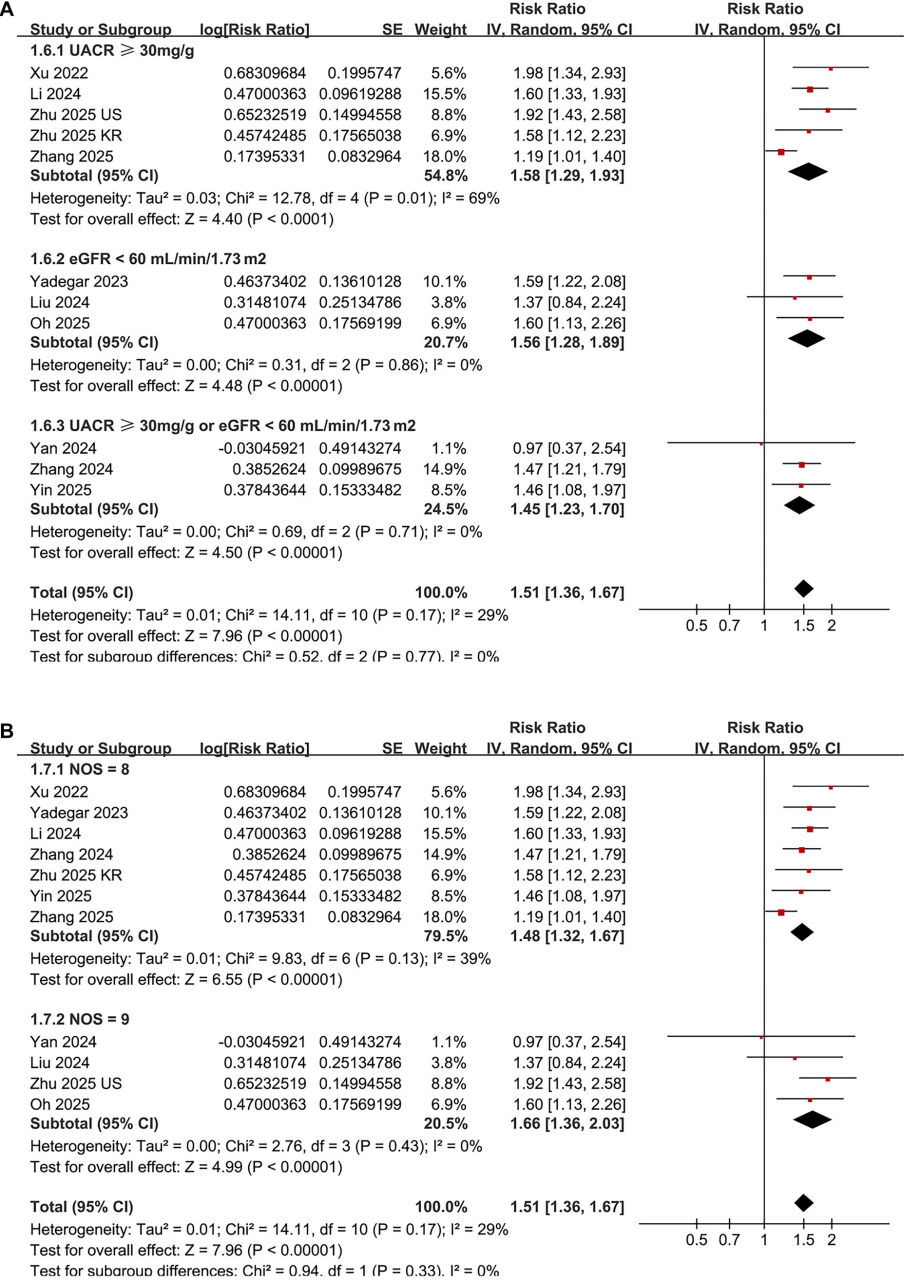
**Subgroup analyses of the association between AIP and DN in patients with T2DM.** (A) Subgroup analysis based on the definition of DN (UACR ≥30 mg/g, eGFR <60 mL/min/1.73 m^2^, or UACR ≥30 mg/g and/or eGFR <60 mL/min/1.73 m^2^); (B) Subgroup analysis based on the Newcastle–Ottawa Scale (NOS) quality scores of included studies (score of 7 vs 8–9). Pooled risk ratios (RRs) with 95% confidence intervals (CIs) are shown for each subgroup. Abbreviations: AIP: Atherogenic index of plasma; DN: Diabetic nephropathy; T2DM: Type 2 diabetes mellitus; UACR: Urinary albumin-to-creatinine ratio; eGFR: Estimated glomerular filtration rate.

Finally, the subgroup analysis indicated a similar association for studies defining DN as UACR ≥ 30 mg/g, eGFR < 60 mL/min/1.73 m^2^, or both (RR: 1.58, 1.56 vs 1.45, *P* for subgroup difference = 0.77; Figure 5A), as well as in studies with NOS scores of 8 or 9 (RR: 1.48 vs 1.66, *P* for subgroup difference = 0.33; Figure 5B).

### Publication bias

**Figure 6.**
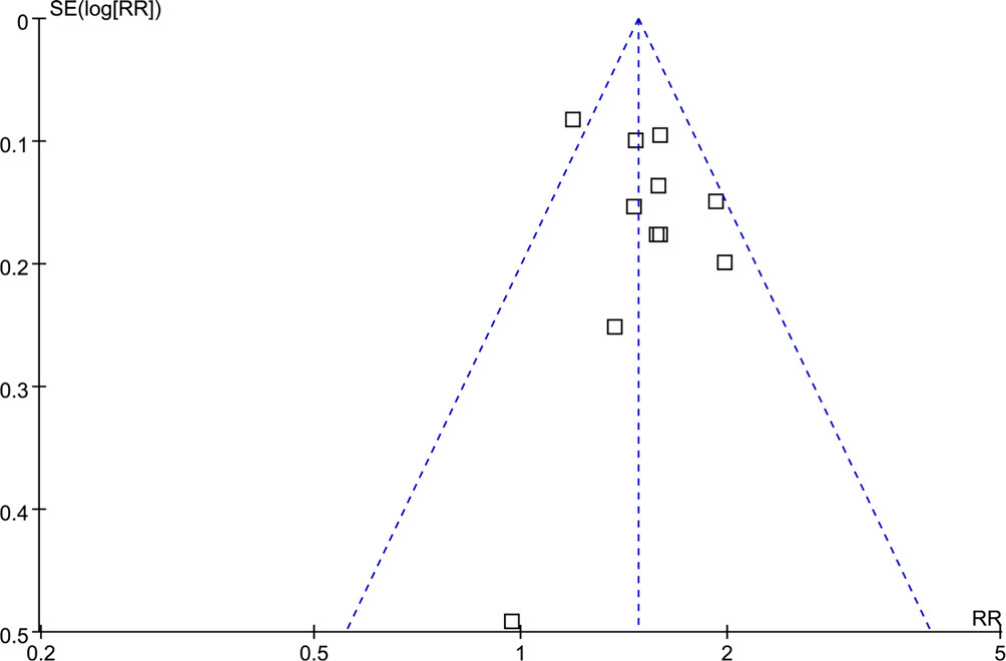
**Funnel plot assessing publication bias in the meta-analysis of the association between high AIP and risk of DN in patients with T2DM.** Visual inspection shows a symmetrical distribution of studies, and Egger’s regression test did not indicate significant publication bias. Abbreviations: AIP: Atherogenic index of plasma; DN: Diabetic nephropathy; T2DM: Type 2 diabetes mellitus.

The funnel plots evaluating the association between AIP and DN in patients with T2DM are presented in Figure 6. A visual inspection of these plots reveals a symmetrical distribution, suggesting a low likelihood of publication bias. This observation is corroborated by Egger’s regression test, which produced a non-significant result (*P* ═ 0.58). Additionally, we conducted Duval and Tweedie’s trim-and-fill analysis to evaluate the potential impact of missing studies. This method did not impute any additional studies, and the pooled effect remained virtually unchanged (RR: 1.51, 95% CI: 1.36–1.67), indicating minimal publication bias.

**Table 1:** Characteristics of the included studies

**Study**	**Country**	**Study design**	**No. of T2DM patients**	**Mean age (years)**	**Mean (%)**	**Methods for AIP cutoff determination**	**Cutoff value of a high AIP**	**Follow-up duration for cohort studies**	**Methods and diagnostic criteria for DN**	**Number of patients with DN**	**Variables adjusted**
Xu, 2022	China	CS	4358	58.7	59.4	Q4:Q1	0.38	NA	UACR ≥ 30 mg/g	1572	Age, sex, duration of diabetes, FBG, insulin, HbA1c, eGFR, serum cystatin C and homocysteine
Yadegar, 2023	Iran	CS	4059	58.5	43.7	Previous study determined	0.24	NA	eGFR < 60 mL/min/1.73 m^2^	657	Age, sex, duration of diabetes, history of hypertension, systolic and diastolic BP, FBG, BMI, and smoking
Yan, 2024	China	CS	4351	53.8	65.3	T3:T1	0.21	NA	UACR ≥ 30 mg/g or eGFR < 60 mL/min/1.73 m^2^	1371	Age, sex, BMI, SBP, hemoglobin, hyperlipidemia, history of CHD, stroke, and concurrent medications
Li, 2024	China	CS	1057	63.4	56.1	Q4:Q1	0.3	NA	UACR ≥ 30 mg/g	464	Age, sex, β2-MG, Fib, D-dimer, FDP, NE, GGT, and FBG
Liu, 2024	China	PC	592	59	54.9	Q4:Q1	0.15	4 years	eGFR < 60 mL/min/1.73 m^2^	53	Age, sex, BMI, SBP, DBP, TC, HbA1c, baseline eGFR, smoking, alcohol drinking, residence, education, and medication use
Zhang, 2024	China	PC	2943	55.2	60.3	T3:T1	0.21	2 years	UACR ≥ 30 mg/g or eGFR < 60 mL/min/1.73 m^2^	709	Age, sex, HbA1c, diabetes duration, BMI, SBP, smoking, drinking, and LDL-C
Zhu, 2025 US	USA	CS	2386	59.5	52.6	Q4:Q1	0.26	NA	UACR ≥ 30 mg/g	418	Age, sex, race, education, BMI, ALT, AST, smoking, alcohol use, hypertension, and CVD
Zhu, 2025 KR	Korea	CS	698	59.9	53.4	Q4:Q1	0.36	NA	UACR ≥ 30 mg/g	135	Age, sex, BMI, ALT, AST, education, smoking, drinking, hypertension, CVD
Yin, 2025	China	CS	683	55.6	67.7	Median	0.34	NA	UACR ≥ 30 mg/g or eGFR < 60 mL/min/1.73 m^2^	390	Age, sex, BMI, hypertension, serum albumin, and Fib
Zhang, 2025	China	CS	3094	56.1	53.2	T3:T1	0.36	NA	UACR ≥ 30 mg/g	676	Age, sex, BMI, waist circumference, DBP, SBP, FBG, HbA1c, LDL-C, TC, SCr, smoking status, alcohol use, history of hypertension
Oh, 2025	Korea	PC	1552	57	61.9	Q4:Q1	0.51	6 years	eGFR < 60 mL/min/1.73 m^2^	489	Age, sex, smoking, alcohol, BMI, SBP, hemoglobin, eGFR, lipid-lowering medication, history of hypertension, CAD, cerebral infarction, dyslipidemia, and fatty liver

For consistency in the meta-analysis, all categorizations of AIP (quartile, tertile, median, or predefined thresholds) were recoded into high-versus-low AIP groups for pooled analysis. Abbreviations: CS: Cross-sectional study; PC: Prospective cohort; AIP: Atherogenic index of plasma; Q1: First quartile; Q4: Fourth quartile; T1: First tertile; T3: Third tertile; UACR: Urinary albumin-to-creatinine ratio; eGFR: Estimated glomerular filtration rate; FBG: Fasting blood glucose; HbA1c: Glycated hemoglobin; β2-MG: Beta-2 microglobulin; Fib: Fibrinogen; FDP: Fibrin degradation product; NE: Neutrophil count; GGT: Glutamyl transpeptidase; SBP: Systolic blood pressure; DBP: Diastolic blood pressure; TC: Total cholesterol; LDL-C: Low-density lipoprotein cholesterol; ALT: Alanine aminotransferase; AST: Aspartate aminotransferase; BMI: Body mass index; SCr: Serum creatinine; CHD: Coronary heart disease; CAD: Coronary artery disease; CVD: Cardiovascular disease; NA: Not applicable.

**Table 2:** Study quality evaluation via the Newcastle-Ottawa scale

**Cohort studies**	**Representativeness of the exposed cohort**	**Selection of the non-exposed cohort**	**Ascertainment of exposure**	**Outcome not present at baseline**	**Control for age and sex**	**Control for other confounding factors**	**Assessment of outcome**	**Enough long follow-up duration**	**Adequacy of follow-up of cohorts**	**Total**
Liu, 2024	1	1	1	1	1	1	1	1	1	9
Zhang, 2024	1	1	1	1	1	1	1	0	1	8
Oh, 2025	1	1	1	1	1	1	1	1	1	9
**Cross-sectional study**	**Adequate definition of cases**	**Representativeness of cases**	**Selection of controls**	**Definition of controls**	**Control for age and sex**	**Control for other confounders**	**Exposure ascertainment**	**Same methods for events ascertainment**	**Non-response rates**	**Total**
Xu, 2022	1	0	1	1	1	1	1	1	1	8
Yadegar, 2023	1	0	1	1	1	1	1	1	1	8
Yan, 2024	1	1	1	1	1	1	1	1	1	9
Li, 2024	1	0	1	1	1	1	1	1	1	8
Zhu, 2025 US	1	1	1	1	1	1	1	1	1	9
Zhu, 2025 KR	1	0	1	1	1	1	1	1	1	8
Yin, 2025	1	0	1	1	1	1	1	1	1	8
Zhang, 2025	1	0	1	1	1	1	1	1	1	8

**Table 3:** Results of univariate meta-regression analysis

**Variables**	**RR for the association between AIP and DN**			
	**Coefficient**	**95% CI**	***P* values**	**Adjusted R^2^**
Mean age (years)	0.018	0.003 to 0.033	0.03	24.3%
Men (%)	0.0064	--0.0175 to 0.0302	0.56	0% (explained heterogeneity < 0)
Cutoff of AIP	--0.022	--1.467 to 1.423	0.97	0% (explained heterogeneity < 0)
NOS	0.13	--0.13 to 0.38	0.29	10.1%

Abbreviations: RR: Risk ratio; AIP: Atherogenic index of plasma; DN: Diabetic nephropathy; CI: Confidence interval; NOS: Newcastle–Ottawa Scale; NA: Not applicable.

### Certainty of evidence

The GRADE assessment rated the overall certainty of evidence regarding the association between high AIP and DN was rated as moderate (Supplemental File 2). This rating is primarily due to the observational design of the included studies, with no downgrading attributed to inconsistency, indirectness, imprecision, or publication bias.

## Discussion

This meta-analysis provides robust evidence supporting a significant association between elevated AIP and DN in patients with T2DM. Our findings indicate that individuals with higher AIP levels have a 51% increased risk of DN compared to those with lower AIP levels. This association was consistently observed across multiple subgroups, and our meta-regression and subgroup analyses identified patient age as a significant modifier, with older individuals demonstrating a stronger association. These results suggest that AIP may serve as a useful marker for identifying T2DM patients at heightened risk of DN, particularly among older adults.

Several pathophysiological mechanisms may explain the observed link between elevated AIP and DN. AIP reflects the balance between triglyceride and HDL-C levels, which in turn indicate the presence of small dense LDL particles, known contributors to atherosclerosis and endothelial dysfunction [35, 36]. Triglyceride-rich lipoproteins promote glomerular injury through lipotoxicity, inflammation, and oxidative stress [37]. Moreover, low levels of HDL-C impair reverse cholesterol transport and reduce the antioxidant and anti-inflammatory capacity of HDL, thereby exacerbating vascular and renal damage [38]. Clinically, dyslipidemia is recognized as a significant contributor to microvascular complications in diabetes [39]. The composite nature of AIP offers a more integrated perspective on lipid-related metabolic disturbances compared to traditional lipid parameters, potentially explaining its stronger association with DN in some studies [12].

The significant impact of age observed in the meta-regression and subgroup analysis may be attributed to age-related changes in vascular integrity and renal autoregulation [40]. Older patients with T2DM typically exhibit longer durations of diabetes, a greater cumulative metabolic burden, and increased susceptibility to both microvascular and macrovascular damage [41]. The age-related decline in renal reserve, coupled with heightened systemic inflammation and dyslipidemia, may exacerbate the negative effects of an elevated AIP on kidney function [42]. Our findings indicate a stronger association in patients with a mean age of 58 years or older, thereby supporting this hypothesis and underscoring the necessity for age-specific risk stratification. Furthermore, it is plausible that older patients have longer diabetes durations, which may contribute to the heightened risk of nephropathy. However, only three studies adjusted for diabetes duration, limiting our capacity to formally assess this effect.

To our knowledge, this is the first meta-analysis to systematically quantify the association between AIP and DN risk in T2DM. The inclusion of a substantial sample size enhances statistical power and facilitates detailed subgroup and sensitivity analyses. The consistent findings across various study settings and analytical methods provide compelling evidence for a robust association. Additionally, the identification of age as a significant effect modifier offers clinically relevant insights into which patient populations may derive the greatest benefit from AIP-based risk assessments.

However, several limitations must be acknowledged. First, all included studies were observational in nature, precluding the establishment of causality between AIP and DN. Second, data were synthesized at the study level rather than at the individual patient level, which may limit the precision of subgroup and meta-regression analyses. Third, although all studies performed multivariate adjustments, residual confounding cannot be completely ruled out. Specifically, the duration of diabetes and the use of lipid-lowering therapies, such as statins, are critical factors that could influence both AIP levels and the development of DN [2]. Unfortunately, these variables were not consistently reported or adjusted for across studies; only three studies accounted for diabetes duration, and few specified or adjusted for statin use. This lack of adjustment may have introduced bias, potentially inflating or attenuating the observed association depending on the direction of confounding. For instance, a longer duration of diabetes is associated with an increased risk of nephropathy and may also affect lipid metabolism, while statin therapy could reduce AIP levels and simultaneously lower DN risk.

The inability to isolate these effects limits our capacity to draw definitive conclusions regarding the independent association between AIP and DN. Future research should incorporate consistent and detailed adjustments for these critical covariates. Additionally, the majority of studies included in our analysis originated from Asian populations, potentially restricting the generalizability of our findings. Variations in genetic backgrounds, dietary habits, lifestyle factors, and access to healthcare services across different populations may affect both AIP levels and susceptibility to DN. Consequently, caution is necessary when extrapolating these results to non-Asian populations, and further validation in more ethnically and geographically diverse cohorts is essential.

Moreover, while we utilized the term DN for consistency, the definition across the included studies often relied on eGFR or albuminuria criteria without histological confirmation. This raises the possibility that some patients with renal impairment not directly attributable to diabetes (e.g., hypertensive nephrosclerosis) may have been included. Furthermore, the studies employed heterogeneous AIP cutoff values (ranging from 0.15 to 0.51) and varied definitions of DN (based on eGFR, UACR, or both), which may compromise direct comparability and introduce outcome misclassification. Although subgroup analyses yielded consistent results across these variations, residual heterogeneity and bias cannot be entirely ruled out.

We also recognize the conceptual limitation of combining cross-sectional (prevalence-based) and cohort (incidence-based) data in a single pooled analysis, as these study designs differ in temporality and may be affected by distinct biases. However, our subgroup and sensitivity analyses demonstrated consistent associations within each design, suggesting that the overall conclusion remains robust despite this methodological heterogeneity.

Finally, we restricted our search to English-language, peer-reviewed publications and excluded grey literature. While this may have resulted in the omission of some non-English or unpublished studies, we aimed to maintain methodological transparency and consistency. The inclusion of only Chinese-language studies could introduce regional bias, and grey literature was excluded due to concerns about methodological reliability and lack of peer review. Despite limiting our search to English-language, peer-reviewed articles, the trim-and-fill analysis indicated no evidence of missing studies, suggesting that the pooled effect is unlikely to be significantly affected by language or publication bias.

Clinically, AIP is an inexpensive and readily available biomarker that can be easily calculated from standard lipid panels [43]. Its application in clinical practice may facilitate the identification of patients with T2DM who are at an increased risk of renal complications, thereby enabling earlier interventions such as intensified glycemic control, lipid management, and nephron-protective therapy [43]. In resource-limited settings, AIP may also serve as a practical alternative to more expensive or less accessible renal biomarkers. However, before AIP can be integrated into routine risk prediction models for DN, further validation through prospective cohort studies across diverse populations is necessary. Future research should focus on elucidating the longitudinal relationship between AIP and the decline in renal function in T2DM, ideally employing standardized definitions and consistent adjustments for potential confounders [44]. Additionally, studies investigating the biological mechanisms underlying the relationship between AIP and DN at a molecular level may uncover new therapeutic targets. Furthermore, interventional trials assessing whether reductions in AIP, achieved through pharmacological or lifestyle modifications, result in improved renal outcomes would provide critical evidence for establishing causality and clinical utility.

## Conclusion

In conclusion, our meta-analysis indicates that elevated AIP is significantly associated with DN in patients with T2DM, particularly among older individuals. While the findings support the potential relevance of AIP in identifying patients at higher risk, the observational nature of the data precludes causal inference. Further prospective studies are needed to validate the association and explore its potential clinical implications.

## Supplemental data

**Supplemental file 1. Detailed search strategy for each database**

**PubMed**

(“Atherogenic Index of Plasma”[Title/Abstract] OR “atherogenic index”[Title/Abstract] OR “AIP”[Title/Abstract]) AND (“Diabetes Mellitus, Type 2”[MeSH] OR “diabetes”[Title/Abstract] OR “diabetic”[Title/Abstract]) AND (“Diabetic Nephropathies”[MeSH] OR “renal”[Title/Abstract] OR “kidney”[Title/Abstract] OR “nephropathy”[Title/Abstract] OR “proteinuria”[Title/Abstract] OR “albuminuria”[Title/Abstract] OR “nephropathies”[Title/Abstract])

**Embase**

(‘atherogenic index of plasma’:ti,ab OR ‘atherogenic index’:ti,ab OR aip:ti,ab) AND (‘diabetes mellitus, type 2’/exp OR diabetes:ti,ab OR diabetic:ti,ab) AND (‘diabetic nephropathy’/exp OR renal:ti,ab OR kidney:ti,ab OR nephropathy:ti,ab OR proteinuria:ti,ab OR albuminuria:ti,ab OR nephropathies:ti,ab)

**Web of Science**

TS=(“atherogenic index of plasma” OR “atherogenic index” OR “AIP”) AND TS=(diabetes OR diabetic) AND TS=(renal OR kidney OR nephropathy OR proteinuria OR albuminuria OR nephropathies)

**Supplemental file 2:** GRADE summary of findings

**Outcome**	**No. of participants (studies)**	**Certainty of the evidence (GRADE)**	**Relative effect (95% CI)**	**Comments**
High AIP vs low AIP for DN risk	25,773 (10 studies)	Moderate	RR = 1.51 (1.36–1.67)	Downgraded for study design (observational); not downgraded for consistency, precision, indirectness, or publication bias. Results were consistent, with moderate heterogeneity and a large effect size

Risk of bias: Downgraded – All included studies were observational in design, which inherently carries a higher risk of residual confounding. Inconsistency: Not downgraded – The results showed low to moderate heterogeneity (*I*^2^ ═ 29%) with consistent effect direction across studies. Indirectness: Not downgraded – The population, exposure, and outcomes were directly relevant to the research question. Imprecision: Not downgraded – The pooled estimate had a narrow 95% confidence interval and large sample size. Publication bias: Not downgraded – Egger’s test and trim-and-fill analysis did not indicate significant publication bias. AIP: Atherogenic index of plasma; DN: Diabetic nephropathy; CI: Confidence interval; GRADE: Grading of recommendations, assessment, development, and evaluation.

## Data Availability

The data that support the findings of this study are available from the corresponding author upon reasonable request.
